# Strong evidence for LncRNA ZNRD1-AS1, and its functional Cis- eQTL locus contributing more to the susceptibility of lung cancer

**DOI:** 10.18632/oncotarget.8411

**Published:** 2016-04-29

**Authors:** Dan Li, Lei Song, Zhongmei Wen, Xiaoping Li, Jing Jie, Yan Wang, Liping Peng

**Affiliations:** ^1^ Department of Respiratory Medicine, The First Hospital of Jilin University, Changchun, China; ^2^ Department of Pediatrics, The First Hospital of Jilin University, Changchun, China

**Keywords:** lung cancer, LncRNA, ZNRD1-AS1, SNP, susceptibility

## Abstract

Long noncoding RNAs (IncRNAs), involved in cancer biology, contributing to essential cancer cell functions such as proliferation, invasion, and metastasis, have received increasing attention recently. Human Zinc ribbon domain containing 1 (ZNRD1) has been confirmed to be involved in carcinogenesis and development of multiple cancers. ZNRD1-AS1, a lncRNA in the upstream region of ZNRD1 which could down-regulate the expression of ZNRD1, has been identified as a possible component in carcinogenesis. The underlying relations of ZNRD1-AS1 with lung cancer development and metastasis remain obscure. In current study, we first evaluated the expression ZNRD1-AS1 and ZNRD1 among lung cancer tissues and corresponding normal tissues, which showed higher expression of ZNRD1-AS1 and lower expression of ZNRD1. To reveal the underlying mechanisms, we then investigated the associations between ZNRD1 eQTLs SNPs in ZNRD1-AS1 and risk of lung cancer in Han Chinese populations. G allele of SNP rs9261204 was significantly associated with an increased risk of lung cancer when compared with A allele (OR: 1.45; 95% CI: 1.19–1.75; *P* = 1.06 × 10^−4^). A weaker, but similar effect was also observed in bladder cancer. SNP rs3757328 was also associated with increased risk of lung cancer (OR: 1.34; 95% CI: 1.07–1.67; *P* = 0.011). Our findings first confirmed the contribution of LncRNA ZNRD1-AS1 to the development of lung cancer in Asian population.

## INTRODUCTION

Zinc ribbon domain containing 1 (ZNRD1) has been confirmed to be involved in carcinogenesis and development of multiple cancers, including gastric cancer, leukemia, esophageal squamous cell carcinoma, renal cell carcinoma, cervical cancer and hepatocellular carcinoma [1–8]. Long non-coding RNAs (LncRNAs) has been confirmed to be associated with susceptibility, invasion, metastasis, and overall and disease-free survival of many cancers [9, 10]. Recently, two studies also evaluated the association of expression quantitative trait loci (eQTL) in LncRNA ZNRD1-AS1, a LncRNA in the upper region of ZNRD1 gene which could down-regulate the gene expression of ZNRD1, with the susceptibility and development of cervical cancer and hepatocellular carcinoma [6, 7]. However, no association study has conducted to evaluate the relationship between gene expression of ZNRD1 and lung cancer up to present, either *in vivo* or *in vitro* studies. Basing on the clues above, we hypothesized that: (1) expression of ZNRD1-AS1 was up-regulated and expression of ZNRD1 was down-regulated in lung cancer tissues; (2) Cis-eQTL loci in LncRNA ZNRD1-AS1 (rs3757328, rs6940552, and rs9261204) contribute to the development of lung cancer. Thus, we conducted this study to test the hypotheses.

## RESULTS

**Figure 1:**
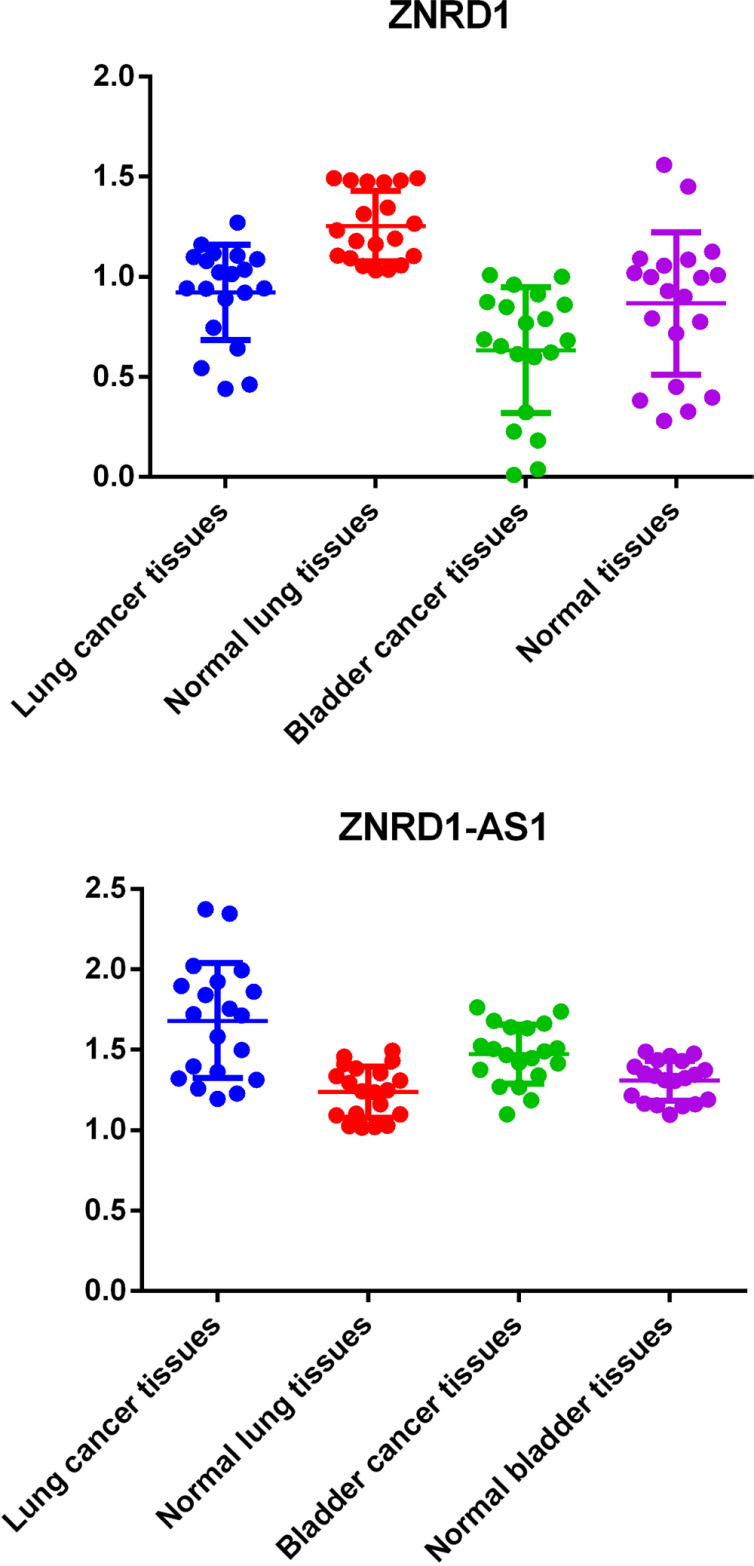
Quantification and statistical analysis of ZNRD1-AS1 and ZNRD1 expression in bladder cancer and matched normal tissues

In current study, we first evaluated the expression levels of ZNRD1-AS1 and ZNRD1 among 20 lung cancer tissues and corresponding normal tissues using Quantitative (real-time) PCR. As shown in Figure 1, the results showed that expression of ZNRD1-AS1 was up-regulated and expression of ZNRD1 was down-regulated in lung cancer tissues (both *P* value < 0.0001). Taken the results above together, these findings suggest a tumor suppressor function for ZNRD1 gene and a tumor contributor function for LncRNA ZNRD1-AS1in the process of carcinogenesis of lung cancer. A weaker, but similar effect was also observed in bladder cancer (Figure 1).

We evaluated the associations of three tagSNPs of the Cis- eQTL for ZNRD1 in ZNRD1-AS1 gene region (rs3757328, rs6940552, and rs9261204) with risk of lung cancer and bladder cancer. As shown in Table 1, the distributions of SNP rs3757328, SNP rs9261204 and SNP rs6940552 in lung cancer and healthy control subjects were displayed. The results showed that compared with A allele, G allele of rs9261204 was significantly associated with 1.45-fold risk of lung cancer (OR: 1.45; 95% CI: 1.19–1.75; *P* = 1.06 × 10^−4^), as well as bladder cancer (OR: 1.33; 95% CI: 1.09–1.61; *P* = 0.004). Per the UCSC Genome Browser, SNP rs9261204 falls within several transcription factor ChIP regions including CTCF, SMC3, RAD21, ZNF143, TEAD4, PBX3, AND FOXA1. Besides, rs3757328 was also associated with 1.34-fold risk of lung cancer (OR: 1.34; 95% CI: 1.07–1.67; *P* = 0.011). Further, rs6940552 showed an marginal association with lung cancer risk (OR: 1.21; 95% CI: 0.99–1.47; *P* = 0.056). Overall, our results provide strong evidence that LncRNA ZNRD1-AS1, and its functional Cis-eQTL locus contribute more to the susceptibility of lung cancer.

**Table 1:** Association between 3 eQTLs SNPs in ZNRD1-AS1 and bladder cancer susceptibility

		Lung cancer		Bladder cancer	
Genotype	Controls	Cases	Adjusted OR (95% CI)*	Cases	Adjusted OR (95% CI)*
**rs3757328**					
GG	340	305	1.00 (reference)	337	1.00 (reference)
AG	150	175	1.30 (0.99–1.70)	146	0.98 (0.74–1.29)
AA	10	20	2.22 (1.05–-4.75)	17	1.71 (0.78–3.77)
**Additive**			1.34 (1.07–1.67)		1.07 (0.85–1.35)
P trend			**0.011**		0.556
**rs6940552**					
GG	289	265	1.00 (reference)	274	1.00 (reference)
AG	169	179	1.15 (0.88–1.51)	175	1.09 (0.84–1.43)
AA	42	56	1.45 (0.94–2.23)	51	1.28 (0.82–1.98)
**Additive**			1.21 (0.99–1.47)		1.13 (0.93–1.38)
P trend			0.056		0.224
**rs9261204**					
AA	280	228	1.00 (reference)	241	1.00 (reference)
AG	180	207	1.41 (1.08–1.84)	200	1.29 (0.99–1.68)
GG	40	65	1.99 (1.30–3.05)	59	1.71 (1.11–2.64)
**Additive**			1.45 (1.19–1.75)		1.33 (1.09–1.61)
P trend			**1.06 × 10^−4^**		**0.004**

* Asjusting for age, gender, smoking status, and drinking status.

## DISCUSSION

In current study, we first examined the gene expression of LncRNA ZNRD1-AS1 and ZNRD1 in lung cancer tissues and corresponding normal tissues; then we investigated whether eQTLs SNPs located in ZNRD1-AS1 and lung cancer risk in Han Chinese populations. Results showed expression of ZNRD1-AS1 was up-regulated and expression of ZNRD1 was down-regulated in lung cancer tissues, and G allele of SNP rs9261204 was significantly associated with lung cancer risk when compared with A allele. Furthermore, a weaker, but similar effect was also observed in bladder cancer. This should be the initial study which aims to explore the relationship between LncRNA ZNRD1-AS1, its variants, and ZNRD1 and development of lung cancer and bladder cancer.

ZNRD1 was downegulated in gastric cancer tissues, compared with adjacent normal tissues [11]. Through transfecting siRNA vectors of ZNRD1 and ZNRD1 cDNA to normal gastric cells or gastric cancer cells, significantly quicker proliferation, and decreased growth rate were detected [11]. In current study, we also found the decreased expression levels of ZNRD1 in the lung cancer tissues, compared with corresponding normal tissues. ZNRD1 spans the HLA region spanning and many studies have suggested that genetic variants of the genes in HLA region contributed to the risk of bladder cancer [12–15]. These evidence above suggested the potential suppressor role of ZNRD1 in carcinogenesis.

IncRNAs, contributing to cancer cell functions including proliferation, apoptosis, invasion, and metastasis, have received increasing attention recently [16–18]. LncRNA ZNRD1-AS1 was located in the upper region of ZNRD1. Wen et al. [7] first reported that rs9261204 and rs6940552 in lncRNA ZNRD1-AS1 may influence not only chronic HBV infection, but also hepatocellular carcinoma (HCC) development. The results of genetic associated study showed that rs3757328 were statistically associated with the lower gene expression of lncRNA ZNRD1 and high gene expression of ZNRD1-AS1 [7]. In current study, SNP rs9261204 was significantly associated with lung cancer and bladder cancer risk. Conclusively, this study showed that LncRNA ZNRD1-AS1, its variants, and ZNRD1 contribute more to the development of lung cancer from different approaches, including *in vivo*, and epidemiological investigations.

## MATERIALS AND METHODS

### Study group and personal interview

Included in this study were 500 newly diagnosed with histologically confirmed lung cancer cases, and 500 controls which were identified among residents of the cases’ neighborhoods of residence and matched to cases by gender, race/ethnicity and age (± 5 years). The exclusion factors for cases included previous cancer, metastasized cancer from other or unknown origin, and previous radiotherapy or chemotherapy. We also replicated the associations among 500 bladder cancer cases. In-person questionnaires administered to all study participants were used to collect demographic variables. A 4 mL peripheral venous blood specimens were collected at the time of interview. Informed consent was obtained from each participant and all procedures and study guidelines were approved by the ethics Committee.

### RNA isolation and qRT-PCR

Total RNA was extracted from cancer tissue samples using TRIzol (Invitrogen, Carlsbad, CA) according to the manufacturer's instructions. The expression levels of ZNRD1-AS1 and ZNRD1 among cancer tissues and corresponding normal tissues were detected using Quantitative (real-time) PCR performed with SYBR Green 2x Master Mix (Life Technologies Carlsbad, CA, USA). Delta CTs were normalized to GAPDH reference gene, and ΔΔCT analysis was performed to calculate relative expression of RNA.

### Determination of eQTL and genotyping

Using database RegulomeDB [19], which guides interpretation of regulatory variants in the human genome, we selected rs3757328, rs6940552, and rs9261204 as the tagSNPs of the Cis- eQTL for ZNRD1 in ZNRD1-AS1 gene region as previously [6, 7]. The genotyping were determined by using a polymerase chain reaction–restriction fragment length polymorphism method (PCR-RFLP). To confirm the genotyping results, PCR-amplified DNA samples were examined by DNA sequencing, and the results were 100% concordant.

### Statistical analyses

First, the expression levels of ZNRD1-AS1 and ZNRD1 between matched pairs of samples were analyzed using Wilcoxon matched-pairs signed rank test. Then, SNP-level association tests for the identified tagSNPs were conducted using unconditional logistic regression treating alternate alleles as an ordinal variable (a log-additive model). Haploview was employed to analyze linkage disequilibrium (LD) parameters (D’ and r2). Statistical analyses were carried out using SAS (Version 9.3; SAS Inc.). *P* < 0.05 in a two-sided test was considered statistically significant.
